# The Association of the Colleges of Dentistry

**Published:** 1867-04

**Authors:** 


### THE ASSOCIATION OF THE COLLEGES OF DENTISTRY.

FROM the official minutes in the present number, it will be seen that this Association has completed its organization. A preliminary meeting was held at Boston last summer, to consult as to its formation. All but the Baltimore College was there represented; and, as was then taken for granted, it has cordially and heartily taken hold of the matter. And now all the Colleges but one—the Pennsylvania College, present a united front, a uniform *curriculum*, a co-operation of action, that can not fail to increase their influence for good. We take it for granted that the Missouri College, by carrying out measures already in contemplation by its corporators, as we are informed, will place itself squarely on the platform of advanced and united Dental education. We are sorry that the brethren of the Pennsylvania College thought it best to go out into the cold, and hope they will reconsider the matter, and come along with us, for we "will do them good." The organization of this Society we regard as the most important step taken by the profession for many years.

